# Delayed diagnosis of traumatic diaphragmatic hernia may cause colonic perforation: a case report

**DOI:** 10.4076/1757-1626-2-6863

**Published:** 2009-08-19

**Authors:** Orhan Veli Ozkan, Ersan Semerci, Ibrahim Yetim, Ramazan Davran, Guvenc Diner, Ilhan Paltaci

**Affiliations:** 1 Department of General Surgery, Mustafa Kemal University, Faculty of MedicineAntakya, HatayTurkey; 2 Department of Radiology, Mustafa Kemal University, Faculty of MedicineHatayTurkey

## Abstract

Early diagnosis of diaphragmatic rupture after traumas may be difficult, and delayed diagnosis may result in increased morbidity and mortality. This paper describes the case of a 32-year-old man who experienced a traffic accident and had diagnosis of traumatic diaphragmatic hernia nearly four months later. The patient was referred to our emergency room suffering from ileus symptoms. Physical examination demonstrated an apparent abdominal distention, tenderness at the upper abdominal quadrants, rebound, and defense. Thoraco-abdominal X-rays and computerized tomography imaging demonstrated intestinal segments with air-fluid levels in thorax. Laparotomy was performed after a preoperative diagnosis of a strangulated-diaphragmatic hernia. At abdominal exploration, it was found that transverse colon and omentum entered into thorax through diaphragmatic defect located at the left diaphragm. Herniating colon segment was complicated with ischemic necrosis and perforation. In conclusion, colon necrosis and perforation may develop when early diagnosis of diaphragmatic ruptures are missed.

## Introduction

Diaphragmatic injury was first reported by Sennertus in 1541 and was repaired successfully by Riolfi for the first time in 1886 [1]. Ruptures of the diaphragm may accompany 0.8-3.6% [2] and 4.5-6% [3] of penetrating or blunt thoracoabdominal trauma and whole body trauma subjects, respectively. Early diagnosis may be difficult. In diaphragmatic ruptures, delayed diagnosis and treatment may result in increased rates of morbidity and mortality [2]. Unfortunately, diagnosis and treatment in patients with diaphragmatic rupture may be delayed as long as several days and even years. Due to late presentation, traumatic events can be forgotten after many years and diaphragmatic injuries can be neglected or omitted [4]. Obstruction and/or strangulation may occur with herniating organs into thorax if an early diagnosis is missed and treatment is not started promptly [5]. This condition can dramatically increase morbidity and mortality rates in such patients[3]. In our study, we presented a case of delayed diagnosis of traumatic diaphragmatic hernia (TDH) with colonic perforation and pulmonary dysfunction.

## Case presentation

**Figure 1.**
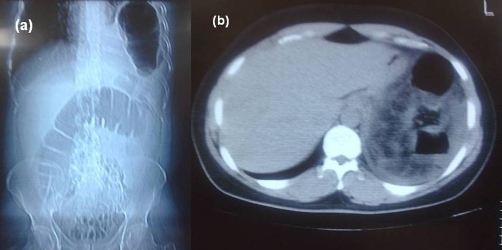
**(a)** X-ray of the abdomen and thorax showed multiple air-filled structures in the left hemithoracic area, widening of the intercostal space and displacement of the mediastinum to the right. Notice the continuity of the colonic gas shadow that can be clearly seen on abdomen and left hemithorax. **(b)** Abdominal CT section showed a thickened posterior diaphragm. Colonic segments can be seen just anterior to the diaphragm.

**Figure 2.**
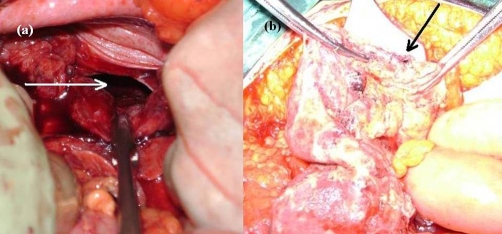
**(a)** Intraoperative view of diaphragmatic rupture. **(b)** Intraoperative appearance of colonic perforation and necrosis.

Herein a 34-year-old Caucasian Turkish male patient who experienced a traffic accident and had the diagnosis of TDH nearly four months later is presented. TDH was secondary to a blunt trauma that resulted from a traffic accident outside the vehicle. After the first trauma, the patient complained of several symptoms such as constipation, shortness of breath which especially increased with effort and pain at upper abdominal quadrants. Nevertheless, those symptoms were increased in severity for the last ten days. The patient was referred to our emergency room suffering from ileus symptoms such as inability to pass gas or stool, abdominal distension, nausea-vomiting and fever. Physical examination demonstrated an apparent abdominal distention, tenderness at the upper abdominal quadrants, rebound and defense. Thoraco-abdominal X-rays (Figure 1a) and computerized tomography (CT) imaging (Figure 1b) had demonstrated intestinal segments with air-fluid levels in the thorax. Laparotomy was performed after a preoperative diagnosis of a strangulated-diaphragmatic hernia. At abdominal exploration, it was found that transverse colon and omentum entered the thorax through a 6-cm diaphragmatic defect located at the left diaphragm (Figure 2a). Celiotomy was employed in hernia and this defect was widened. Transverse colon and omentum then returned into the abdomen. The herniating colon segment was complicated with ischemic necrosis and perforation (Figure 2b). The omentum wrapping around the colonic defect has appeared to be restricting further thoracic invasion by the intestinal content. Partial resection of colon, end-to-end anastomosis and partial omental resection were performed due to necrosis. The diaphragmatic defect was repaired with nonabsorbable sutures. Postoperatively, the patient developed a wound infection, which immediately resolved with drainage and antibiotic treatment. The patient was discharged on the postoperative sixth day.

## Discussion

Mechanism of an injury due to a blunt trauma can be explained by lateral impact that leads to distortion of chest wall, and direct frontal impact resulting in increased intra-abdominal pressure; thus both causing a diaphragmatic injury and visceral herniation. Although blunt trauma may cause an injury at any of the part of the diaphragm, most injuries occur in the postero-lateral aspect of the left side of the diaphragm. Traumatic diaphragmatic injuries may be due to blunt (68-75%) and penetrating (25-32%) traumas. Diaphragmatic injuries mostly occur in young men and left-sided hemidiaphragmatic injuries are more common (56-90%) [5]. This patient was 34-year-old and had a medical history of a blunt trauma due to a traffic accident and experienced an isolated diaphragmatic injury.

In case of TDH, abdominal organs may herniate into the thorax due to pressure difference between the peritoneal space and thorax. As the diameter of the diagraphmatic injury increases in time, herniating of the abdominal organs and subsequent clinical symptoms ensue. Herniating organs into thorax make a pressure on the lungs and lead to mediastinal shift. Therefore, some patients may be referred to hospital complaining of cardiac and respiratory symptoms. Herniating organs may be related with a number of additional symptoms and signs such as gastrointestinal distress and ileus [4]. Dyspnea due to pressure on lungs was apparent in our patient. He was presented with inability to pass gas or stool, nausea-vomiting, fever secondary to colon strangulation and perforation.

There is a common consensus that radiological studies may be helpful to diagnose traumatic diaphragmatic hernias. Nevertheless, there are some restrictions in the diagnostic modalities to diagnose traumatic diaphragmatic injuries. It may be difficult to diagnose a traumatic injury both in early and late cases and therefore it is very important to suspect a traumatic injury condition. In a study, Shah et al., showed that the disorder was diagnosed preoperatively in 43.5% of 980 cases, but the diagnosis of 41.3% of the patients was performed during autopsy or surgical exploration, and in the remaining 14.6% of the patients, diagnosis was delayed [6]. A careful physical examination and auscultation of the lungs, various methods of investigation such as chest X-ray, direct abdominal graph at a standing position and fluoroscopy and barium studies, intraperitoneal contrast injection, magnetic resonance imaging and ultrasonography, computed tomography, diagnostic laparoscopy can be helpful for the diagnosis. Occasionally, laparotomy may be the only route of diagnosis. Although a chest X-ray is a frequently applied diagnostic method, it has been shown to be preoperatively successful in only half of the cases [7]. Chest X-ray sensitivity in detecting diaphragmatic injuries for the left-side injuries is 46% and 17% for the right [8]. Ultrasound guided preoperative successful diagnosis is achieved in 71% of the patients. The diagnosis by CT for a left-sided blunt trauma and a right-sided injury is made in 78% and 50% of the subjects respectively. Stomach construction and herniation of the abdominal organs, diaphragmatic discontinuity on CT imaging suggest the diagnosis of diaphragmatic injury. CT sensitivity and specificity for the diagnosis of acute traumatic diaphragmatic ruptures are 61-71% and 87-100%, respectively [9]. Our patient was diagnosed by an abdomino-thoracic X-ray and computerized tomography demonstrating multiple air-filled structures in the left hemithoracic area, widening of the intercostal space and displacement of the mediastinum to the right, continuity of the colonic gas shadow that can be clearly seen on abdomen and left hemithorax, a thickened posterior diaphragm, and colonic segments seen just anterior to the diaphragm.

A surgical intervention can be carried out for a TDH through the abdominal or thoracic route. Abdominal organs may have changed their locations toward the thorax. When evaluating an abdominal trauma and organs located in the abdomen, an abdominal approach should be considered. Delayed TDH may require a thoracic approach to ease the release of adhesions in the thorax. The thoracic approach is preferable in patients with a thorax trauma, a large defect in the diaphragm and accompanying emphysema. If necessary, an abdominal exploration can be conducted by a thoraco-abdominal incision [10].

However, abdominal approach can be more convenient in case of abdominal organ injury. TDH must be restored by non-absorbable sutures or synthetic grafting. In our case, there was a delay of 4 months. Preoperative physical examination, thoracoabdominal X-ray and computerized tomography showed a herniating colon into the thorax. Consequently, colon resection was performed by abdominal approach and restored successfully with nonabsorbable sutures. However, there was no need for synthetic grafting for the repair of hernia. The present case was complicated with wound infection. The absence of complications related with the thorax such as atelectasis was attributed to the omental wrapping of the colonic perforation site.

In conclusion, a history of trauma must be investigated to diagnose the patients with a delayed TDH condition due to a blunt trauma. An unnoticed TDH must be kept in mind. A careful examination of the thorax X-ray and computerized tomography imaging may be helpful. Colon necrosis and perforation may ensue when the early diagnosis is missed.

## Abbreviations

CT: computerized tomography
TDH: traumatic diaphragmatic hernia
